# Perception of enhanced learning in medicine through integrating of virtual patients: an exploratory study on knowledge acquisition and transfer

**DOI:** 10.1186/s12909-024-05624-7

**Published:** 2024-06-11

**Authors:** Zhien Li, Maryam Asoodar, Nynke de Jong, Tom Keulers, Xian Liu, Diana Dolmans

**Affiliations:** 1 Department of Educational Development & Research, School of Health Professions Education, Faculty of Health, Medicine and Life Sciences, Maastricht University, Maastricht, Netherlands; 2 School of Health Professions Education, Department of Health Services Research, Faculty of Health, Medicine and Life Sciences, Maastricht University, Maastricht, Netherlands; 3 Department of Radiation Oncology (Maastro), GROW School for Oncology and Reproduction, Maastricht University Medical Centre, Maastricht, The Netherlands

**Keywords:** Virtual patient, Medical education, Role modeling, Feedback, Authentic cases

## Abstract

**Introduction:**

Virtual Patients (VPs) have been shown to improve various aspects of medical learning, however, research has scarcely delved into the specific factors that facilitate the knowledge gain and transfer of knowledge from the classroom to real-world applications. This exploratory study aims to understand the impact of integrating VPs into classroom learning on students’ perceptions of knowledge acquisition and transfer.

**Methods:**

The study was integrated into an elective course on “Personalized Medicine in Cancer Treatment and Care,” employing a qualitative and quantitative approach. Twenty-two second-year medical undergraduates engaged in a VP session, which included role modeling, practice with various authentic cases, group discussion on feedback, and a plenary session. Student perceptions of their learning were measured through surveys and focus group interviews and analyzed using descriptive statistics and thematic analysis.

**Results:**

Quantitative data shows that students highly valued the role modeling introduction, scoring it 4.42 out of 5, and acknowledged the practice with VPs in enhancing their subject matter understanding, with an average score of 4.0 out of 5. However, students’ reflections on peer dialogue on feedback received mixed reviews, averaging a score of 3.24 out of 5. Qualitative analysis (of focus-group interviews) unearthed the following four themes: ‘Which steps to take in clinical reasoning’, ‘Challenging their reasoning to enhance deeper understanding’, ‘Transfer of knowledge ‘, and ' Enhance Reasoning through Reflections’. Quantitative and qualitative data are cohered.

**Conclusion:**

The study demonstrates evidence for the improvement of learning by incorporating VPs with learning activities. This integration enhances students’ perceptions of knowledge acquisition and transfer, thereby potentially elevating students’ preparedness for real-world clinical settings. Key facets like expert role modeling and various authentic case exposures were valued for fostering a deeper understanding and active engagement, though with some mixed responses towards peer feedback discussions. While the preliminary findings are encouraging, the necessity for further research to refine feedback mechanisms and explore a broader spectrum of medical disciplines with larger sample sizes is underscored. This exploration lays a groundwork for future endeavors aimed at optimizing VP-based learning experiences in medical education.

**Supplementary Information:**

The online version contains supplementary material available at 10.1186/s12909-024-05624-7.

## Introduction

In Medical Education, a persistent challenge lies in the bridge between acquiring theoretical knowledge and applying it in real-world clinical scenarios. Many medical students struggle with translating their classroom learning into practical settings. The primary challenge lies in effectively translating the concepts students have learned into authentic patient interactions. This gap is particularly concerning because it affects the quality of patient care, as medical students are not just learning to acquire knowledge but must be able to apply this knowledge in complex healthcare settings.

One approach to address this challenge is the use of Virtual Patients (VPs), a computer-based simulation of real-life clinical scenarios for students to train clinical skills [1]. Research has shown that using VPs in the classroom can effectively improve various aspects of learning, from core knowledge and clinical reasoning to decision-making skills and knowledge transfer [2–5]. The VPs provide students with the opportunity to practice skills in a safe and controlled simulation environment.

Recent studies have focused on optimizing the design and arrangement of VPs as part of learning activities to facilitate both knowledge acquisition and retention [6–8]. For instance, Verkuyl, Hughes [8] demonstrated that using VPs as gamification tools can improve students’ confidence, engagement, and satisfaction.

However, studies focusing on the specific factors that contribute to these improvements when integrating VPs into the classroom are limited, particularly in understanding how to use VPs in the classroom to facilitate the transfer of knowledge students’ gain from the class to the subsequent studying stage of their education and eventual practice.

Acquisition and transfer of knowledge are critical factors in medical education, as medical students must be able to apply their knowledge and skills to real-world clinical scenarios [9]. Research suggests that for the effective transfer of knowledge, students should be immersed in authentic environments, enabling the transition of learned competencies to advanced stages [10–13].

Despite the consensus on the efficacy of VPs as a tool, there is a gap in understanding how to integrate VPs in the classroom to optimize students’ learning, especially in facilitating learning transfer. The effectiveness of VPs is not just in their use but also in how they are used by students to enhance their understanding on how to reason and make decisions about medical treatments when dealing with clinical cases. Without a clear and deep understanding, we risk underutilizing their potential and losing opportunities for medical students to become well prepared for real-world clinical scenarios.

**Fig. 1:**
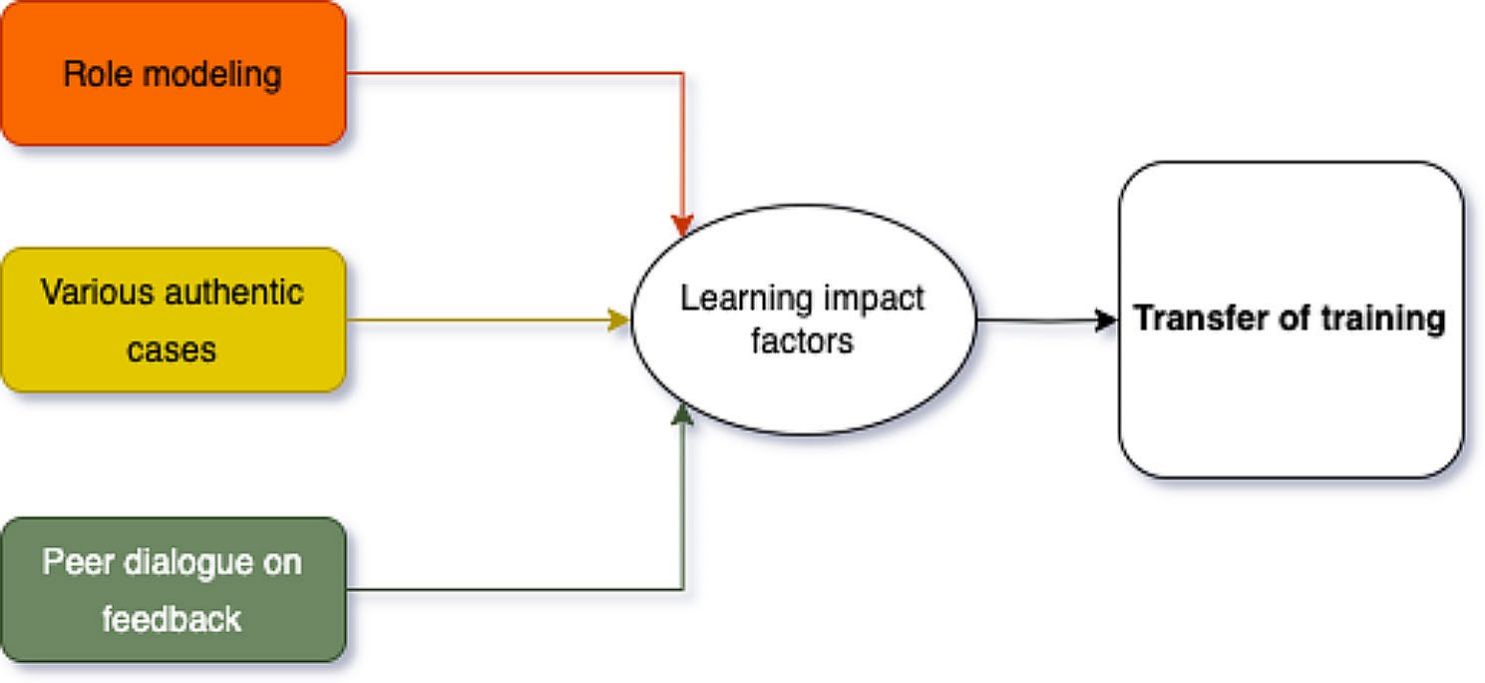
Relationship of implementing, impact factor, and transfer of training

Certain elements, such as role modeling instruction [14–16], using various authentic cases [17–19], and engaging in peer discussions on feedback [20–22], emerge as potential key components that could be integrated to maximize the knowledge acquisition via VPs. For instance, Stalmeijer, Dolmans [23] show how an expert, serving as a role model, provides guidance that facilitates student learning by demonstrating clinical skills and reasoning out loud. While there is ample evidence supporting the advantages of inclusion of VPs in education, there is not enough research focusing on the detailed aspects of effective instructional design techniques. This paper delves into these components, seeking to understand how the VP integration influences students’ learning and knowledge transfer. Figure 1 shows the theoretical framework of how integrating VPs in class affects students’ learning and might impact the transfer of learning in a simulated VP environment to practice.

This exploratory study aims to investigate how instructional design elements such as role modeling, various authentic cases, and peer dialogues on feedback within VP sessions affect students’ learning from the learner’s perceptions. The core research question in this study focuses on how the implementation of role modeling, various authentic cases, and peer dialogue on feedback in VPs, influences learners’ perception of knowledge gain and transfer in personalized medicine.

## Method

### Setting

The study was conducted at Maastricht University in the elective course, “Personalized Medicine in Cancer Treatment and Care”. This course is open to second-year undergraduate medical students of Maastricht University.

### Participants

Initially, 24 students enrolled in this course for the academic year of 2022–2023, and 22 students participated in the Virtual Patient session. In total, 19 students voluntarily completed the survey designed to evaluate their experiences and perceptions of the Virtual Patients session. Thereafter, 9 of the 19 survey respondents voluntarily agreed to participate in three focus group interviews, with 2–4 students in each focus group. Students were informed that participation in this research study had no impact on student’s academic performance or their continuation in their studies.

### Intervention

**Fig. 2:**
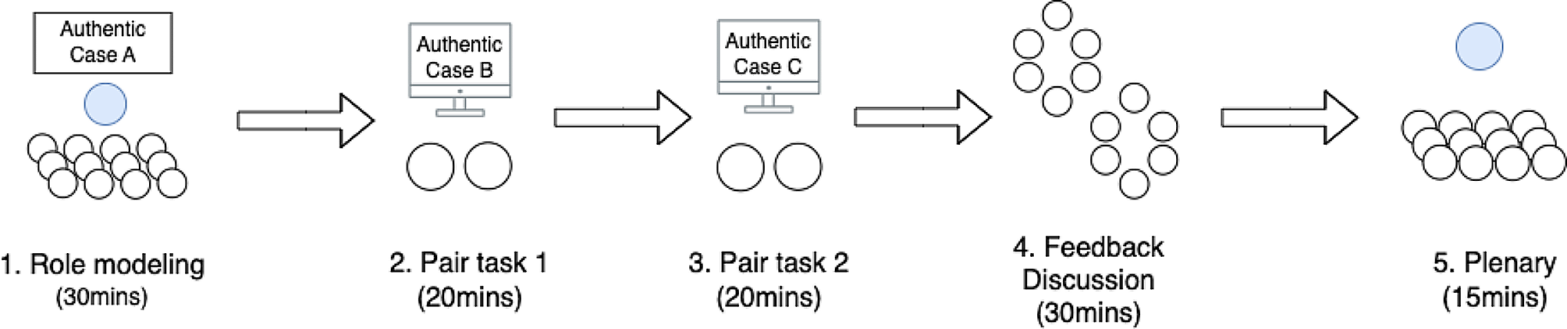
The flow of integrated virtual patient session

The instructional approach for the VP cases was structured in a specific format for the students. Figure 2 shows the instructional design for VP integration. The first stage was a role-modeling phase, where an expert demonstrated the clinical reasoning process using VP Case A. This was followed by a practice session where students worked in pairs on two different VP cases (Case B and C). After that, students formed two larger groups each including 5 or 6 students, and discussed the system feedback that was provided by VP platform. Finally, the expert summarized the session and addressed students’ questions. The whole intervention lasted 120 min. Figure 1 gives an overview of the intervention steps.

1. Role modeling (30 min): The intervention started with an expert, a clinician with teaching experience, demonstrating a clinical case (Case A) and showing the clinical reasoning process by thinking aloud. The expert served as a role model in showcasing the approach toward clinical problem-solving, provided supportive information, and demonstrated how to proceed through the case. The aim of the role modeling session was to empower students to apply the insights and methodology gained from experts in case A to solve subsequent cases (case B and case C), Although these cases shared similarities in underlying principles, they diverged on patient characteristics such as age, complications, and smoking history that can influence patient treatment outcomes.

2 and 3. Two VP pair tasks (20 min each): In this segment, the 22 participating students were paired, resulting in 11 pairs. These pairs were then divided into two groups. Group 1 (6 pairs) and group 2 (5 pairs) alternated in going through Case B and Case C to account for the practice effect. These cases were variations of the clinical cases introduced during the role-modeling demonstration, differing in patient characteristics such as age, complications, and smoking history to challenge the students’ reasoning. Students were encouraged to work collaboratively.

4. Feedback discussion (30 min): Upon completion of the VP cases, an automated feedback is immediately provided about the reasoning analysis. Participants were instructed to save this feedback for later discussion. After that, Students were organized into groups of six, based on the sequence in which they engaged with the cases. For instance, those who first practiced with Case B and then proceeded to Case C formed Group (1) Conversely, students who started with case C and then moved on to case B were assembled into Group (2) To foster meaningful dialogue, students engaged in discussions focused on the feedback generated by the Virtual Patient system, guided by a printed discussion guide distributed to each group (see Appendix 2). The discussion aimed to deepen students’ understanding and enrich their conversations about the cases they had just completed.

5. Plenary (15 min): This part lasted 15 min. Hosted by the expert to summarize the session and address questions or doubts raised by students.

During the practice and discussion sessions, the expert circulated among the groups to offer additional guidance and support.

### The virtual patient cases

Three Virtual Patient (VP) cases (Case A, B, and C) were created to enhance students’ comprehension of specific concepts, knowledge, and skills in clinical reasoning. The VP practice was developed on the P-Scribe (www.pscribe.nl) learning platform, a web-based e-learning system based in the Netherlands. The platform facilitates the design and implementation of text-based VP sessions (Appendix 4).

While these cases shared a foundation on authentic head and neck cancer treatment, they were characterized by varying patient characteristics in terms of age, gender, and medical history (anamnesis).

**Fig. 3:**
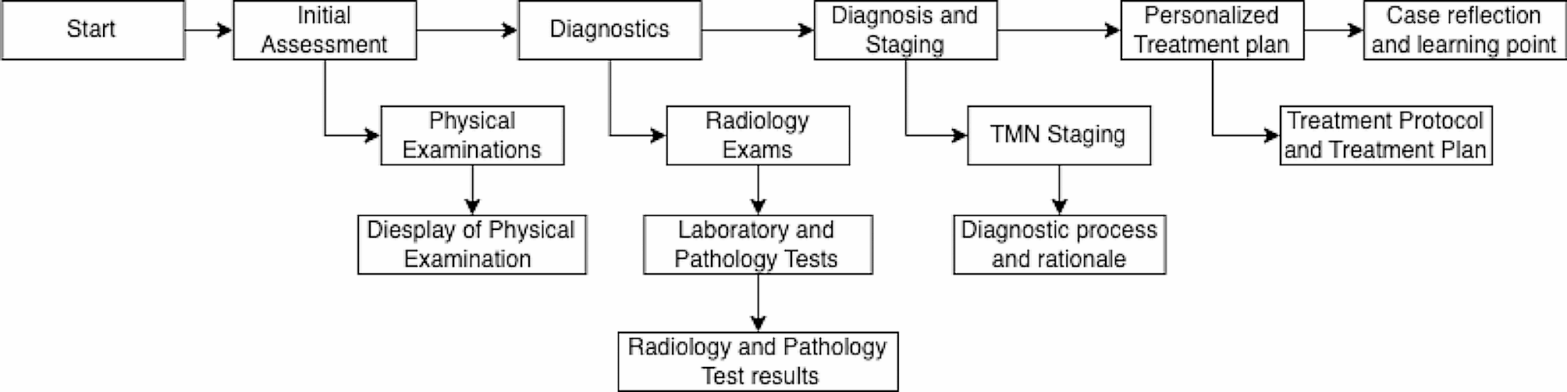
VP case flow chart

Within each VP case, students were presented with a scenario related to neck cancer. Figure 3 shows the chart of a VP case. Each case starts with an overview of the patient and their medical history which students had to use to make an initial assessment. After this, students encountered a mix of multiple-choice and open-ended practice questions. These questions guided students in planning diagnostics, formulating a diagnosis, and devising a treatment plan tailored to the patient’s specific needs. Immediate feedback was provided after students submitted each response, and comprehensive summative feedback was given at the conclusion of each case to foster understanding and learning from any potential misjudgments or oversights (See Appendix 4).

### Measurement instruments

*Learning-perception survey*: The survey (Appendix 1) consisted of 20 items, structured into five primary sections: general experience, intended learning outcome, role modeling, practicing with various authentic cases, and reflection on peer dialogue around feedback. The first item asked about students’ general experience through the whole session. The second item focused on their perception of intended learning outcomes. Six items then focused on the students’ perceptions of learning through role modeling followed by 5 items addressing perceptions related to their learning on practicing with authentic cases. The final seven items explored students’ perception of learning from dialogue around feedback. Participants indicated their level of agreement for each statement using a 5-point Likert scale: 1 denoting “Strongly Disagree”, 2 for “Disagree”, 3 for “Neutral”, 4 for “Agree”, and 5 for “Strongly Agree”. For interpretation, average scores below 3 were considered as “in need for improvement”, those of 4 or higher as ‘good’, and those between 3 and 4 as ‘neutral’.

*Focus group interviews*: Three focus group interviews (Appendix 3) were conducted to dive deeper into students’ perceptions of their learning experience, knowledge gain, and knowledge transfer in real-world settings. The focus group took place after the survey and the survey data did not affect the development of the focus group questions. In focus group 1, two students, in focus group 2, two students and in focus group 3, five students participated. The interviews were structured around a series of questions that explored students’ perceptions of their learning across specifically designed sections. These sections included Role Modeling, Practice with Various Authentic Cases, and Dialogue around Feedback. The structure aimed to understand students’ perspectives on each key component of the learning sections.

### Analysis

The analysis of the survey data was conducted by calculating the mean, standard deviation, and the Alpha Coefficient for the responses pertaining to each of the five key dimensions of the survey. The mean score provided an indicator of the average student perception, while the standard deviation offered insights into the variability of the responses. The Alpha Coefficient, a measure of internal consistency, was computed to assess the reliability of the survey dimensions. Through these statistical measures, an overall understanding of the students’ perceptions regarding the various aspects of the Virtual Patients was attained, facilitating a robust analysis aligned with the research objectives.

The focus-group interview data were analyzed following the thematic analysis procedure set out by Braun and Clarke [24]: (1) familiarize yourself with your data, (2) generate initial codes, (3) search for themes, (4) review themes, (5) define and name themes, and (6) produce the report. The interview was guided by pre-existing frameworks or theories in medical education. This ensured the capture of major aspects of the VP learning experience as underscored in the existing literature: role modeling, using various authentic cases, and peer dialogue around feedback [16–18, 20, 21]. The focus group interview was recorded, transcribed, and coded by three team members and ordered in initial themes (Z.L, M.A, and X.L). These themes were discussed with the larger team. We used a process of inductive and deductive analysis and used the three design principles of role modeling, practice with various authentic cases, and group discussion on feedback as sensitizing concepts to study the data [24]. Thereafter, quantitative and qualitative analyses were collectively appraised, compared, and checked for inconsistencies. In this triangulation, the themes identified in focus-group interviews were explanatory to the descriptive statistics of the survey.

### Trustworthiness

Several measures were taken to enhance the study’s trustworthiness. First, triangulation was achieved by employing multiple data collection methods, including surveys and focus group interviews. The interview data collection continued until saturation was reached, ensuring a comprehensive understanding of the student’s experiences and perceptions. Secondly, the coding process followed an iterative approach. Team members initially coded transcripts independently, and then met to reach a consensus before moving on to code subsequent transcripts. Three researchers conducted the coding independently to minimize bias and enhance the validity of the findings. Finally, a member check among a sample of the focus group interviewees was conducted. In response to the question asking whether they agreed with summaries of preliminary results and would provide comments, confirmatory responses were received as well as some minor additional comments and clarifications. The latter were taken into account in the analysis and interpretation of the data.

### Ethical approval

The Maastricht University Ethical Committee reviewed and approved this study. The approval number is FHML-REC/2023/021.

## Results

The findings from both the survey data and focus group interviews were presented to explore students’ perceptions of the effectiveness of the Virtual Patient (VP) Session in enhancing their clinical reasoning skills.

### Survey data

The survey explored students’ perceptions across five key dimensions: General Experience, Intended Learning Outcome, Role Modeling, Practicing with Various Authentic Cases, and students’ reflection on Peer Dialogue around Feedback. The students scored the VP sessions on 20 items (Table 1). The scores varied between M = 2.95 to M = 4.58, on a scale of 1–5.

**Table 1:** Survey results of students’(*n* = 19)

Factors	Mean	SD	Alpha Coefficient
**General Experience of Virtual Patient Session (Q1-Q2)** Q1. My overall experience of the Virtual Patient session is positive. Q2. The Virtual Patient session helps me improve my clinical reasoning skills.	4.13 4.11 4.16	0.70	
**Students’ Perception of learning from Role Modeling Session (Q3-Q8)** Q3. The demonstration by the experienced clinician at the start of the session enhanced my understanding of the intended learning outcomes of the Virtual Patient session. Q4. The demonstration by the experienced clinician at the start of the session was useful in guiding me when going through the Virtual Patient cases myself. Q5. The demonstration by the experienced clinician at the start of the session was useful for gaining a better understanding on how to reason when dealing with a similar patient case. Q6. The reasoning-out-loud approach used by the clinician when going through the clinical case at the start enhanced my understanding of the reasoning behind the choices made when going through the clinical case. Q7. The clinician demonstrated the specific clinical steps that are necessary to know when going through a Virtual Patient case. Q8. The demonstration of the clinician at the start stimulated me to adopt a similar approach when working with the Virtual Patient cases myself.	4.38 4.58 4.32 4.58 4.47 4.32 4.26	0.61	0.80
**Students’ Perception of learning from Practicing with Authentic Cases (Q9-Q13)** Q9. The provided two Virtual Patient cases fitted well with my current level of understanding. Q10. Engaging with the two Virtual Patient cases enhanced my understanding of the subject matter. Q11. Engaging with the two Virtual Patient cases enhanced my understanding of the complexities inherent in real-world clinical scenarios. Q12. Discussing similarities and differences between the two Virtual Patient cases helped me to better understand variations in treatment approaches between different patients. Q13 Engaging with these virtual patients will enable me to apply what I have learned to real clinical practice.	4.00 3.89 4.37 3.74 3.95 4.05	0.86	0.66
**Perception of learning from Peer Dialogue around Feedback (Q14-Q20)** Q14. The peer dialogue on feedback enhanced my understanding of the subject matter. Q15. The feedback provided by the Virtual Patient system is constructive. Q16. The feedback provided by the Virtual Patient system enhanced meaningful discussion in our group. Q17. The peer dialogue on feedback was effective in helping me understand the feedback provided by the Virtual Patient system Q18. The peer dialogue on feedback will enable me to take what I have learned into real practice Q19. The peer dialogue on feedback helped me generate specific strategies to address the feedback provided by the Virtual Patient. Q20. The peer dialogue helped me prioritize the areas I still need to improve.	3.24 3.16 3.89 3.26 3.26 2.95 3.00 3.16	1.05	0.85

For the General Experience of Virtual Patient Session (Items Q1-Q2) the average score was M = 4.13 (SD = 0.70). Specifically, the overall experience was positively rated at M = 4.11. The component that assessed the improvement of clinical reasoning skills received an average score of M = 4.16.

Regarding the Students’ Perception of Learning from Role Modeling (Items Q3-Q8), the average score was M = 4.38 (SD = 0.61). Students agreed that the expert demonstration at the start of the session helped them understand the intended learning outcomes and was useful in guiding them through the Virtual Patient cases, with scores ranging from M = 4.26 to M = 4.58.

Students’ perception of learning from practicing with various authentic cases (Items Q9-Q13), received an average score of M = 4.00 (SD = 0.86). The scores measured the students’ perception of how well the provided Virtual Patient cases matched their current level of understanding, enhanced their comprehension of the subject matter, and helped them grasp the complexities inherent in real-world clinical scenarios.

For their perception of learning from Peer Dialogue around Feedback (Questions 14–20), the average score was M = 3.24 (SD = 1.05). These scores measure the students’ perception of the effectiveness of peer dialogue in enhancing understanding, generating strategies to address feedback, and prioritizing areas of improvement.

### Focus group interview data

The interviews revealed five themes: ' Which steps to take in clinical reasoning’, ' Asking challenging questions to enhance deeper understanding of knowledge’, ‘The variety in cases helps to enhance transfer to the real world’, and ‘Deeper understanding of reasoning through reflections’.

### Which steps to take in clinical reasoning

Students acknowledged the expert’s initial demonstration helped them to develop structured knowledge and gain understanding of the clinical reasoning process.

*I think it (Role modeling) helps to find a pattern in clinical reasoning as well. At first, it (the expert) explained to us. For example, are there possible lymph nodes? Yes or no. Then you need to do this and this…Then you can make kind of…pattern that differs for the diagnosis and the prognosis. So you can make kind of a diagram in your head. Which you can use later on. And your knowledge becomes more structured. (Focus Group 2, Student B)*

Students also perceived that the integrated practice with Virtual Patients helped them to anticipate the subsequent steps in clinical reasoning. They indicated the patterns learned through practicing with virtual Patients helped them understand the procedures they needed to follow to evaluate the patient.

*I think now I know the steps which they (the procedural) followed to evaluate the patient, so first we can do this and then that. First, you determine the TNM (Tumour, Node, Metastasis) staging and do the endoscopy, then the TNM staging, and then you make the treatment plan. Now it’s more clear how they do those steps. (Focus Group 1, Student A)*

Moreover, students thought the pair work and dialogue helped them think and clarify with each other what steps they needed to do in clinical reasoning when they had different opinions.

*Yeah, that (pair working) was really nice because you can discuss, like I think do this and the other one says, you know, I think do that step, and then you’re already discussing the answers which is really nice to have. (The discussion) really make you think about the steps. (Focus Group 1, Student b)*

### Challenging their reasoning to enhance deeper understanding

Students reported how the course design differed from other blocks. According to the students, the VP practice was particularly beneficial in helping them integrate knowledge, and make the knowledge their own.

*It (the VP practice) helps you to integrate knowledge because other blocks are really only lectures, they are all listening and listening. So the virtual patient was really nice to make this stuff our own. (Focus Group 2, Student A)*

Students indicated the examples given by the expert helped them get a better understanding of the more detailed TNM (Tumor, Node, Metastasis) table, that are used in clinical reasoning.

*Yeah, she (the expert) gave examples and guided the reading of the tables for TNM (Tumor, Node, Metastasis) staging, and those were also in the Virtual Patient cases, but because she already used them once and explained how we have to use them, it became more clear to us, what these tables are for and how they are used (Focus Group 1, Student B)*.

The students noted that in VP practice sessions, compared with passive learning in traditional lectures, they were challenged to engage directly with the material by making clinical decisions, such as selecting appropriate tests to reach a diagnosis.

*In lectures, we passively learn the trajectory from symptoms to diagnosis. During Virtual Patient practice, we actively process it. So you have to make decisions and select the test etc. (Focus Group 2, Student B)*

Students indicated that practicing with the VP cases challenged them to look up information and reasoned by themselves. They gave an example of the imaging practice in which they were tasked with examining specific body parts in medical images on their own, they thought they were challenged to reason about what they saw instead of getting the information directly.

*Yeah, also the (medical) imaging in the assignments where you need to look at a specific part of the body, normally you just see a picture and someone says, yeah, this is the stomach or this is the heart, whatever, and now you need to look it up yourself and think about it yourself, what you see, so that really helps. (Focus Group 1, Student B)*

Furthermore, they emphasized the questions asked by experts challenged them to think, put the knowledge in their own words and apply the knowledge with their own reasoning.

*The questions she (the expert) asked really make you think about the things she’s learning(teaching). So if she asks questions, you’re really thinking, and yeah, you’re challenged to put it in your own words. (Focus Group 1, Student B)*

*For instance, she (the expert) asked questions that not from official guidelines, instead, it came from where widely doctor worked and her personal experiences. I applied what she said with my own reasoning behind it. (Focus Group 2, Student B)*

### Transfer of knowledge

Students perceived that practicing with VP cases in different situations offered them hands-on experience, where they actively engaged with various situations, which prepared them for future patient interactions.

*Having cases that are closer to the real world, like the comorbidity we discussed, would make it more realistic. (For instance, ) What if he also has obesity or diabetes? Those are the patients that we are going to see in the future. So it helps out a lot to have those different conditions as well. (Focus Group 2, Student B)*

Students also indicated their preference for the structured approach of the VP session, where an initial demonstration by an expert, sharing their clinical experience, followed by hands-on practice with VP cases was perceived to enhance transfer to practice. This method, as described by the student, bridged the gap between theoretical knowledge and practical application. They think this structure made the knowledge clear and further helped them to transfer their knowledge from theory to practice.

*You (the Virtual Patient session that integrated with role modeling, authentic VP practice, and peer discussion around feedback) made it (the clinical reasoning) clear for me because of the first case we discussed with the teacher. Well, he discussed it and showed us how to think, and how to get things from certain perspectives with risk factors, age, et cetera. And then we do it ourselves. We had to find out what was wrong and go on. So I quite liked it. It gave me a deeper understanding. (Focus Group 3, Student A)*

Students indicated the sense of practical immersion is amplified by the “side information that you don’t really need” *(Focus Group 3, Student E)* from the cases. They highlighted the side information represented the interaction with real patients and made them think of clinical situations in real-world settings.

*(Side) information would be more realistic, also side information that you don’t really need because a patient also tells you a lot of things, and some of those things aren’t as important, but you still need to decide if they are important or not. What do you see, why do you see it, what’s different than normal. (Focus Group 3, Student E)*

Moreover, several students indicated that the hypothetical “what-if” discussions during the role modeling session helped them with reasoning, prompting them to consider complications that might arise in real-life medical situations.

*So for example, about age, it’s more difficult to do a treatment above 70. (What if that patient) has things like smoking history and that kind of stuff. I think it’s really valuable because you have already had an example about it (Demonstrating Case A). (Focus Group 1, Student A)*

Students indicated that the diagnosis practice in VP led them to realize the difference in real-world scenarios. They said while in the simulated environment might seem easy to choose multiple diagnostic options, in the real world, medical professionals must make more selective decisions due to limitations. They think this experience taught them to think of prioritizing and decision-making in a realistic medical setting.

*Yeah, maybe also there (in VP cases) were also a question about which imaging techniques you would use and then it was Echo or CT, MRI, there was also an option where you could listen to the lungs and some of the people also checked that one, but it isn’t really necessary, so you think it only takes one minute, so why not, but in the real world there isn’t always time to do everything, so it’s also good to think what is really necessary and what’s not. (Focus Group 1, Student A)*

### Enhance reasoning through reflections

During the VP session, students received feedback and conducted conversations around the feedback provided by the Virtual Patient system. Students thought the peer dialogues around feedback provided opportunities for collective reflection and insights, allowing them to pinpoint areas of improvement.

*I thought that (the peer dialogue) was really useful, because sometimes one person, for example, when the teacher explains everything, you don’t pick up everything he says. She (your peer) might pick up a different thing, and I pick up a different thing, and we can ask each other, do you know how this works? So I thought that was really useful. (Focus Group 3, Student B)*

The students emphasized the importance of expressing and discussing different opinions. They noted that such interactions could provide new insights and perspectives that they would not have considered independently, thereby enriching their understanding.

*When you do have different opinions, I think they (your peers) can give you insight that you maybe didn’t have for yourself. So you can add to each other’s knowledge. If somebody has another view, then we can discuss it. It (the discussion) brightens my tunnel view. Also having to say it (the knowledge) out loud and explaining your thoughts to someone else can also help, I think. (Focus Group 2, Student A)*

When talking about the peer dialogues around feedback during the VP session, Some students highlighted the benefits of immediate feedback, which provided them with clarity and instant validation. However, others saw value in delayed feedback, as it fostered discussion and multiple interpretations.

*I liked that the Virtual Patient program, that it gave you immediate feedback. That was really handy. And I also liked the discussion afterward so we could speak about it a bit more (Focus Group 3, Student B)*.

*There was immediate feedback on most questions, so you knew if you had been correct or wrong. But for the learning process it might be handy to have that after the group discussion, because now we all have the same answer. (Focus Group 2, Student B)*

## Discussion

The study demonstrated the perception of students’ learning and knowledge transfer by integrating VP cases with role modeling introductions, and peer dialogue around feedback, specifically in the context of personalized medicine in cancer treatment and care. The survey reflected a positive learning experience and students reported they gained a better understanding of the clinical reasoning process as well as which steps to take when dealing with a clinical case through this specific course design with integration of VP cases. Qualitative data showed that the integration of VPs into the educational setting clearly shifted the students from being passive observers in a traditional lecture-based format to active participants in a simulated clinical environment. This shift is in line with previous research findings, which suggest that the use of VPs in clinical training actively engages learners and encourages the application of their knowledge [4].

The quantitative data revealed that students highly valued the role modeling session, as indicated by the high average scores. Qualitative data explained that the role modeling session enabled students to not only observe the clinical process being demonstrated but also to engage in active thinking by interacting with the expert. As discussed by Cruess, Cruess [15], role modeling not only consciously imparts knowledge but also unconsciously influences students’ attitudes and behaviors, making the learning experience more relatable to the clinical environment. In this study, by sharing clinical reasoning and personal anecdotes during the class, experts made the learning experience more relatable to the clinical environment that students would face in the future. This mirrored the *role modeling research* by Morgenroth, Ryan [25] which emphasizes the importance of role models in shaping the self-concept and motivation of individuals. Moreover, the qualitative data showed that the demonstration by the expert serves as a fundamental pre-knowledge for students to cover the knowledge gap and prepare them with the following practice. This finding aligns with van Merrienboer’s scaffolding concept emphasizing the importance of initial expert guidance in learning processes [16].

Followed by the role modeling demonstration, students practiced on two VP cases in pairs and perceived that the VP practice enhanced their clinical reasoning skills, and also helped them understand the real-world clinical setting. The result showed that the variety and real-life complexity of cases in the VP sessions were perceived to be essential for students’ knowledge gain and transfer. The positive perception of various authentic cases aligns with previous research highlighting the importance of exposure to diverse and authentic scenarios in medical training [17, 18]. Moreover, the hypothetical “what-if” scenarios further enhanced students’ analytical abilities, preparing them for the multifaceted challenges they would encounter in real-world medical situations. Survey responses (Q10, mean = 4.37; Q13, mean = 4.05 in Table 1) indicated a consensus among students on the improvement with this practice in understanding and applying knowledge. Our findings corroborate with Jonassen and Hernandez-Serrano [26]’s study emphasis on the importance of authentic learning environments for effective knowledge transfer.

After the practice, students discussed the feedback provided by the VP system. Despite its mixed quantitative reception, the peer dialogue on feedback was qualitatively found to be a vital component for promoting critical thinking, discussion, and reflection. The Feedback from the VPs, both immediate and delayed, along with peer dialogue, emerged as crucial elements in students’ learning process. In this study, students showed different preferences for receiving feedback. Some students preferred immediate feedback, however, others preferred delayed feedback. How feedback was provided notably influenced peer interactions. Given that immediate feedback was dispensed upon submission of answers, the peer dialogues automatically started when students noticed disparities or encountered obstacles. Such dialogues not only served to resolve ambiguities but also fostered collective reflection, enhancing comprehension of the subject. By vocalizing their thoughts and engaging in active discussions, students were able to solidify their understanding and uncover nuances they might have missed otherwise. This aligns with the importance of engaging in peer discussions on feedback as outlined in the theoretical background [20–22].

When looking at the integration of VP cases with the particular course design, students perceived that the expert demonstration, followed by VP practice, and peer dialogue around feedback fostered a comprehensive understanding, allowing them to integrate diverse clinical knowledge, which in turn promoted understanding. The “Watch-think-do-reflect” structure not only ensured better knowledge retention but also enhanced students’ enthusiasm towards the subject. Observing model demonstrations enabled students to assimilate clinical nuances and contemplate real-world applications. Subsequent hands-on practice with VP cases fortified their cognitive structures, honing their clinical reasoning. Ultimately, students perceived that reflective peer discussions on feedback solidified their learnings, enhancing knowledge retention.

### Limitations

This study employed a survey and focus group interviews that provided a comprehensive understanding of students’ perceptions of learning. However, there are several limitations. The study had a small sample size and was conducted in the context of an elective course, which may limit the generalizability of the findings. Furthermore, the study was exploratory in nature and did not measure actual learning outcomes or long-term retention, which are critical aspects of educational impact.

### Implications for future research

Future research should investigate whether integrating Virtual Patients (VPs) into classroom activities enhance student learning outcomes by incorporating learning assessments and involving larger and more diverse participant groups to validate our findings. Additionally, a deeper analysis of students’ reasoning processes and interactions could provide insights into how and why knowledge gain and transfer are fostered or hindered. Furthermore, it is also important to understand the most beneficial moment for integrating VPs into educational settings to enhance transfer from a simulated to a real practice setting. This understanding could inform the development of more effective educational strategies and interventions.

## Conclusion

The integration of Virtual Patients into classroom learning appears to offer a promising approach to enrich medical education. Key elements such as role modeling and various authentic cases contribute positively to students’ perception of learning, as well as peer dialogue on feedback. However, the approach to peer dialogue on feedback may need to be refined for more consistent benefits. Furthermore, studies with larger sample sizes and broader participant groups are essential to provide robust support for the efficacy of this educational approach and its components.

### Electronic supplementary material

Below is the link to the electronic supplementary material.

Supplementary Material 1

Supplementary Material 2

Supplementary Material 3

Supplementary Material 4

## Data Availability

The datasets used and/or analysed during the current study are available from the corresponding author on reasonable request.
