# Supplementary material for: Perception of enhanced learning in medicine through integrating of virtual patients: an exploratory study on knowledge acquisition and transfer
Source: BMC Med Educ. 2024 Jun 11;24:647. doi: 10.1186/s12909-024-05624-7 (PMC11165759; doi:10.1186/s12909-024-05624-7)
Supplement: Supplementary file 1 — Supplementary Material 1 [file 12909_2024_5624_MOESM1_ESM.docx]

# Appendix 1

# Survey

**Demographic Info:**

| **Gender** |  |
| --- | --- |
| **Age** |  |

*Thank you for participating in our survey about your experience with the Virtual Patient Session. The purpose of this survey is to gather feedback on your perception of learning in this Virtual Patient Session in the personalized medicine in cancer treatment and care course. Your responses will help us improve our Virtual Patient sessions and better understand how they can facilitate learning in this subject area. Thank you for taking the time to provide your feedback.*

1. Have you read the information letter and do you consent to participate in this research?
   1. Yes
   2. No

**Answer the following question on a scale of 1-5 (1=Strongly disagree, 2=disagree, 3=neutral, 4=agree, 5= strongly agree)**

1. My overall experience of the Virtual Patient session is positive.

2. The Virtual Patient session helps me improve my clinical reasoning skills.

## You started the session seeing a demonstration of the first case by an experienced clinician…

1. The demonstration by the experienced clinician at the start of the session enhanced my understanding of the intended learning outcomes of the Virtual Patient session.

2. The demonstration by the experienced clinician at the start of the session was useful in guiding me when going through the Virtual Patient cases myself.

3. The demonstration by the experienced clinician at the start of the session was useful for gaining a better understanding on how to reason when dealing with a similar patient case.

4. The reasoning-out-loud approach used by the clinician when going through the clinical case at the start enhanced my understanding of the reasoning behind the choices made when going through the clinical case.

5. The clinician demonstrated the specific clinical steps that are necessary to know when going through a Virtual Patient case.

6. The demonstration of the clinician at the start stimulated me to adopt a similar approach when working with the Virtual Patient cases myself.

## After the demonstration by the clinician, you engaged in working on two Virtual Patient cases yourself...

1. The provided two Virtual Patient cases fitted well with my current level of understanding.

2. Engaging with the two Virtual Patient cases enhanced my understanding of the subject matter.

3. Engaging with the two Virtual Patient cases enhanced my understanding of the complexities inherent in real-world clinical scenarios.

4. Discussing similarities and differences between the two Virtual Patient cases helped me to better understand variations in treatment approaches between different patients.

5 Engaging with these virtual patients will enable me to apply what I have learned to real clinical practice.

## After the engagement with the two Virtual Patient cases yourself, you followed up with a discussion on the feedback provided by the Virtual Patient system…

1. The peer dialogue on feedback enhanced my understanding of the subject matter.

2. The feedback provided by the Virtual Patient system is constructive.

3. The feedback provided by the Virtual Patient system enhanced meaningful discussion in our group.

4. The peer dialogue on feedback was effective in helping me understand the feedback provided by the Virtual Patient system

5. The peer dialogue on feedback will enable me to take what I have learned into real practice

6. The peer dialogue on feedback helped me generate specific strategies to address the feedback provided by the Virtual Patient.

7. The peer dialogue helped me prioritize the areas I still need to improve.

Would you be willing to participate in an interview about your experiences? If so, can you give me your email address below _________________
