# Supplementary material for: Perception of enhanced learning in medicine through integrating of virtual patients: an exploratory study on knowledge acquisition and transfer
Source: BMC Med Educ. 2024 Jun 11;24:647. doi: 10.1186/s12909-024-05624-7 (PMC11165759; doi:10.1186/s12909-024-05624-7)
Supplement: Supplementary file 2 — Supplementary Material 2 [file 12909_2024_5624_MOESM2_ESM.docx]

# Appendix 2

# Group Discussion Guide

This guide is intended to facilitate your group discussion on the feedback provided by the Virtual Patient system after your practice session. Engaging in these discussions will help you better understand the feedback, identify areas for improvement, and build strategies for better performance in future sessions.

# Step 1: Prepare for the Discussion (8min)

1. Review the Feedback: Take the time to read through the feedback provided by the Virtual Patient. Understand your strengths and areas for improvement.
2. Reflect on Your Performance: Think about your performance during the Virtual Patient practice. What did you do well? What could you have done differently?
3. Identify Discussion Points: Note down specific points from the feedback that you would like to discuss. These could be points you found particularly helpful, confusing, or surprising.

# Step 2: During the Discussion (20min)

1. What physical examinations did you decide to conduct? What findings led you to these choices and how did the results inform your next steps?
2. Which radiology and pathology tests did you opt for, and why? Why are other options wrong?
3. Describe your treatment plan for this patient. How did you ensure it was personalized for this specific patient? What are the factors that determine the personalized treatment plan? Why did you decide not to choose other treatment options that were available?
4. After seeing the feedback at the end of the PScribe provided by an expert, which part of your approach do you think you have to change and why??
5. Compared with the two other cases, did you find any significant similarities or differences between the two cases?
6. What are your key learnings from this exercise and how will you apply these in your future practice?
7. What areas do you feel you need more practice or learning in, based on this experience?
