# Supplementary material for: Perception of enhanced learning in medicine through integrating of virtual patients: an exploratory study on knowledge acquisition and transfer
Source: BMC Med Educ. 2024 Jun 11;24:647. doi: 10.1186/s12909-024-05624-7 (PMC11165759; doi:10.1186/s12909-024-05624-7)
Supplement: Supplementary file 3 — Supplementary Material 3 [file 12909_2024_5624_MOESM3_ESM.docx]

# Appendix 3

# Interview question:

## General Questions:

1. Can you describe your experience with the Virtual Patient session?

2. Were there any specific aspects of the sessions that stood out to you as particularly beneficial for learning? And why?

3 Were there any specific aspects of the sessions that stood out to you as particularly challenging for learning, And why?

*Thank you. The virtual patient session has been designed based on certain educational ideas and principles. I would like to elaborate on this with you.*

## Role-modeling:

1. We started the session with a demonstration by an experienced clinician. How did this enhance (or hinder) your learning? Why so? Please explain and give examples.

2. Can you provide examples of specific approaches you adopted from the demonstration and used when working with the Virtual Patient cases yourself? Explain why it was helpful.

## Various authentic cases:

1. After the demonstration by the clinician, you engaged in working on two virtual cases yourself. In what ways and why did working with the cases in the Virtual Patient sessions help you develop a better understanding of the complexities of real-world clinical scenarios (or not)? Can you give examples and explain why.

2. In what ways and why did a discussion about similarities and differences between the cases enhance or hinder you to gain a better understanding of the complexities of real clinical scenarios or not?

## Peer Dialogue on Feedback:

After the engagement with two cases yourself, you followed up with a peer dialogue on the feedback provided by the system.

1. Can you explain how the peer dialogue on feedback during the Virtual Patient session enhanced or hindered your learning and why so?

2. What aspects of the peer dialogue on feedback during the Virtual Patient session did you find most valuable and why so?

3. At the end of the session, can you describe what is the personalized factor in that case and what is the same for all the cases?
