# Supplementary figures and images for: Perception of enhanced learning in medicine through integrating of virtual patients: an exploratory study on knowledge acquisition and transfer
Source: BMC Med Educ. 2024 Jun 11;24:647. doi: 10.1186/s12909-024-05624-7 (PMC11165759; doi:10.1186/s12909-024-05624-7)

**Appendix 4**


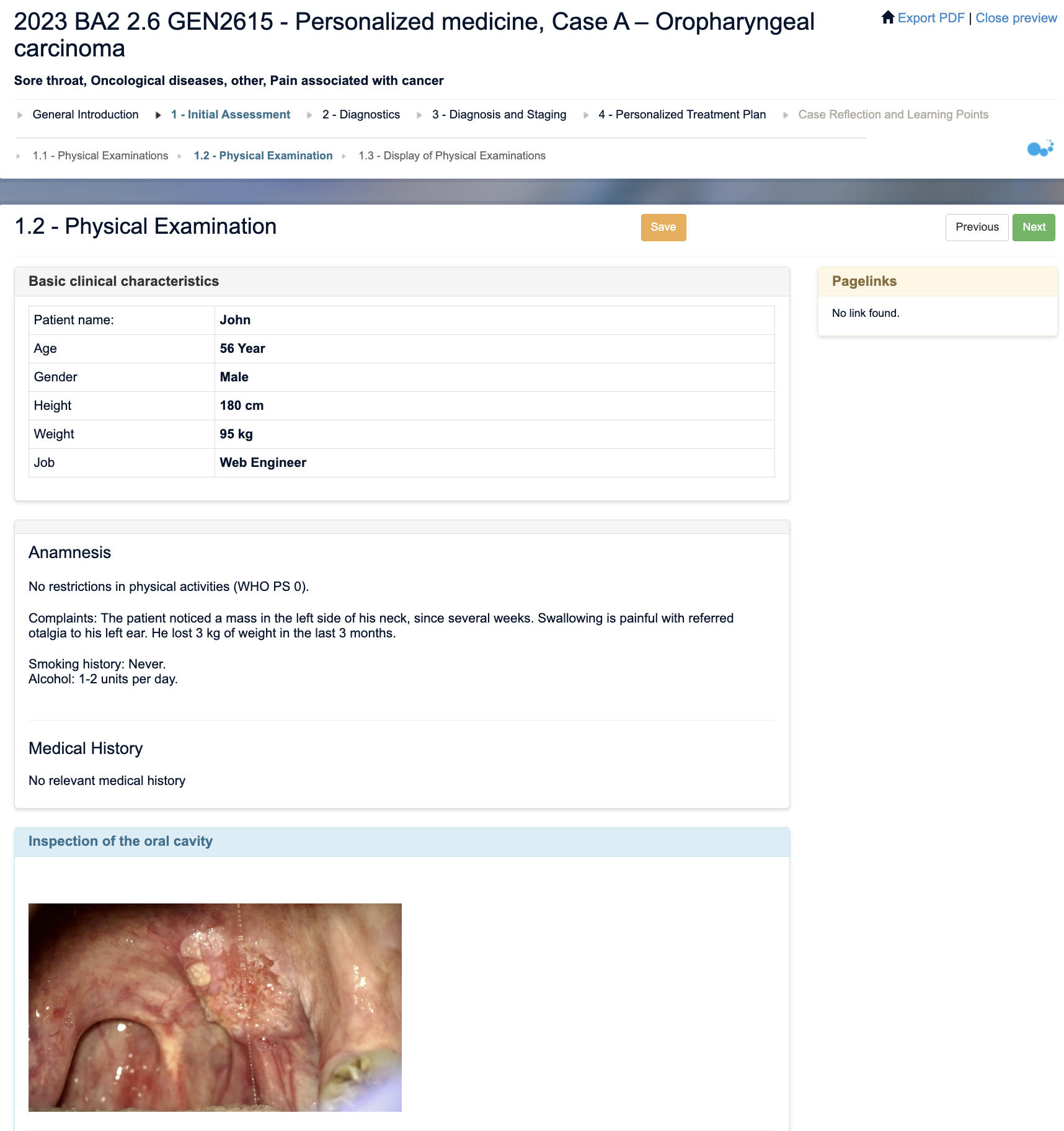


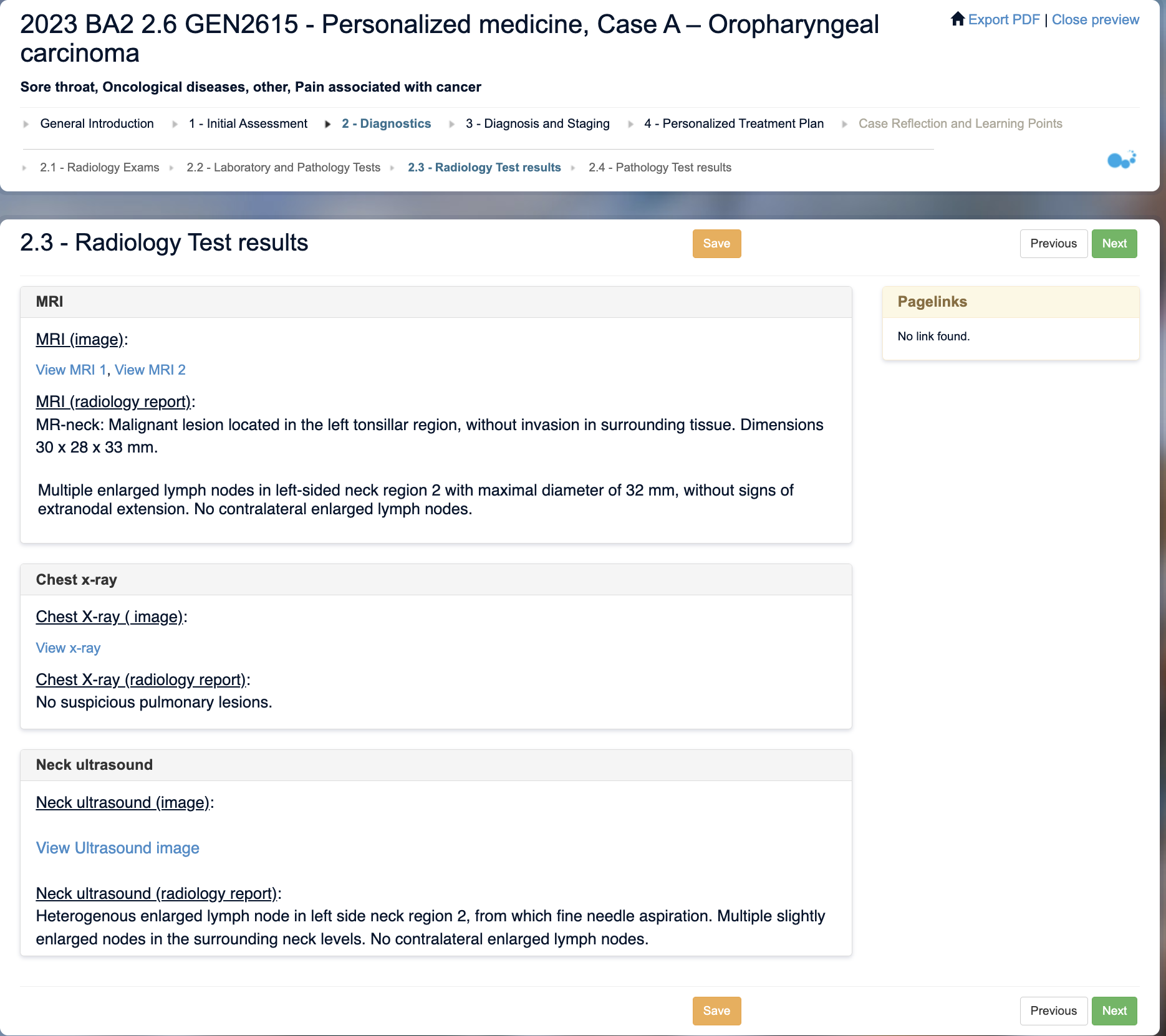


Virtual Patient Interface and Feedback


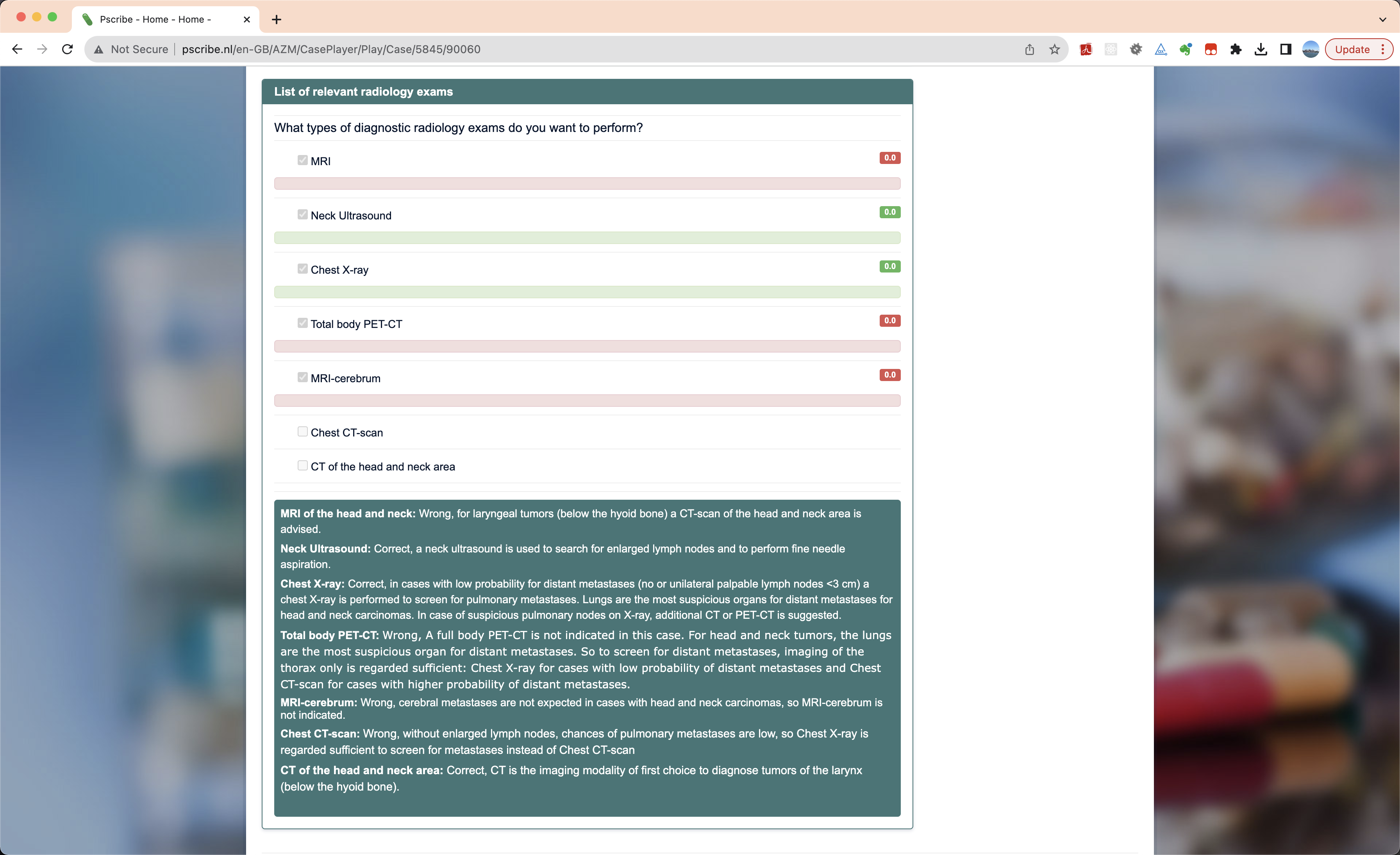


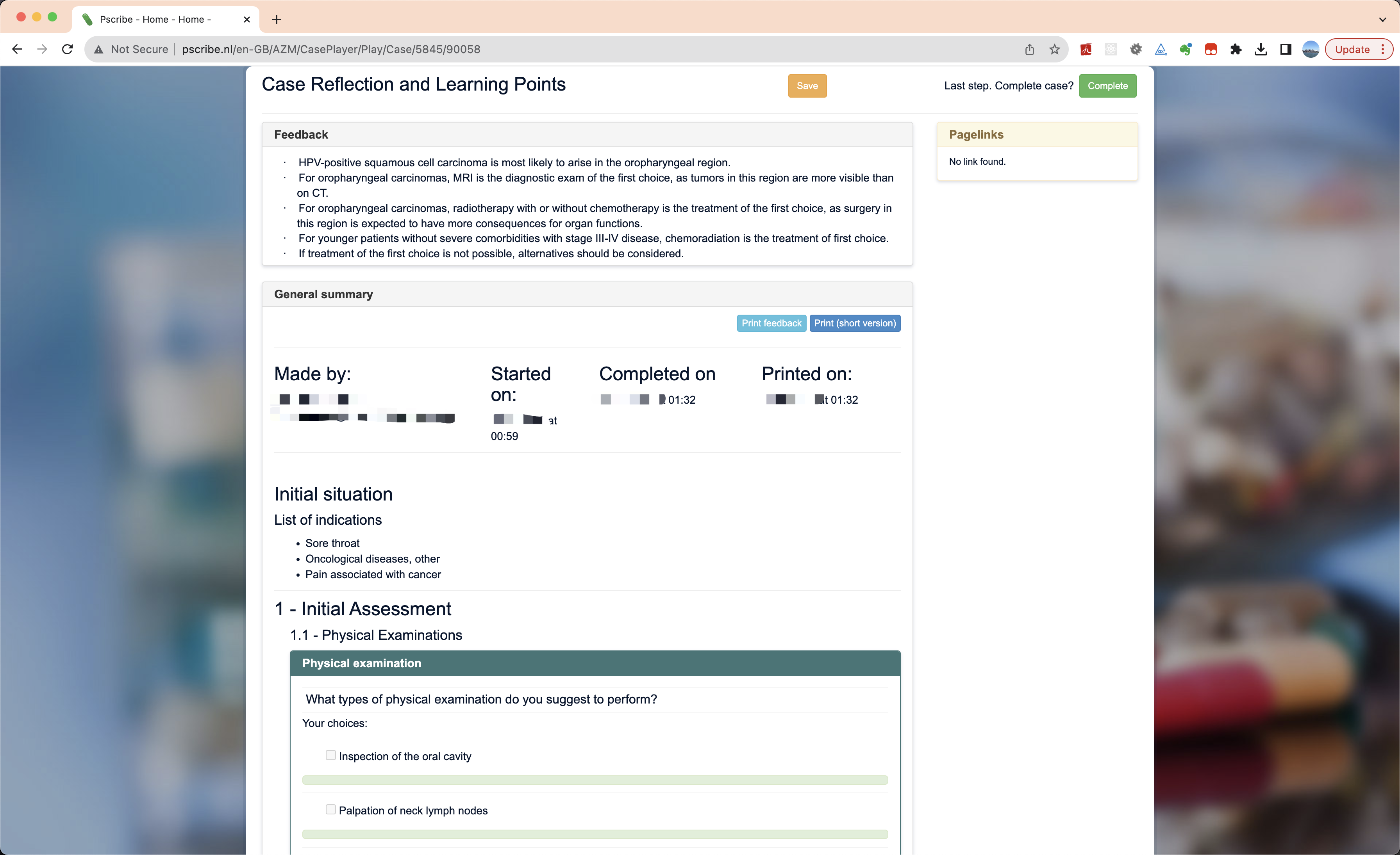

Supplement: Supplementary file 4 — Supplementary Material 4 [file 12909_2024_5624_MOESM4_ESM.docx]
